# Targeting Oral Biofilms: Comparative In Vitro Evaluation of Commercial Dental Antiseptics Against Clinical and Reference Microbial Strains

**DOI:** 10.3390/ijms27083450

**Published:** 2026-04-12

**Authors:** Vanessa Bolchis, Delia Abrudan-Luca, Ramona Dumitrescu, Atena Galuscan, Marioara Nicoleta Caraba, Ion Valeriu Caraba, Roxana Popescu, Mihaela Adina Dumitrache, Gabriela Ciavoi, Daniela Jumanca

**Affiliations:** 1 Translational and Experimental Clinical Research Centre in Oral Health, Faculty of Dental Medicine, “Victor Babes” University of Medicine and Pharmacy Timisoara, 300041 Timisoara, Romania; vanessa.bolchis@umft.ro (V.B.); dumitrescu.ramona@umft.ro (R.D.); galuscan.atena@umft.ro (A.G.); jumanca.daniela@umft.ro (D.J.); 2 Clinic of Preventive, Community Dentistry and Oral Health, Faculty of Dental Medicine, “Victor Babes” University of Medicine and Pharmacy Timisoara, 300041 Timisoara, Romania; 3 Department of Cellular and Molecular Biology, Faculty of Medicine, “Victor Babeș” University of Medicine and Pharmacy Timisoara, 300041 Timisoara, Romania; 4 ANAPATMOL Research Center, “Victor Babeș” University of Medicine and Pharmacy Timisoara, 300041 Timisoara, Romania; valeriucaraba@usvt.ro (I.V.C.); popescu.roxana@umft.ro (R.P.); 5 Faculty of Bioengineering of Animal Resources, University of Life Sciences “King Mihai I” from Timisoara, 119 Calea Aradului, 300645 Timisoara, Romania; 6 Oral Health and Community Dentistry Department, Faculty of Dental Medicine, “Carol Davila” University of Medicine and Pharmacy, 17-21 Calea Plevnei Street, Sector 1, 010221 Bucharest, Romania; mihaela.dumitrache@umfcd.ro; 7 Department of Dental Medicine, Faculty of Medicine and Pharmacy, University of Oradea, 410073 Oradea, Romania; gciavoi@uoradea.ro

**Keywords:** dental antiseptics, chlorhexidine, ozone, antibacterial agents, dental plaque

## Abstract

Oral biofilms are complex polymicrobial communities involved in the development of dental caries and periodontal diseases. Chemical antiseptics are commonly used as adjuncts to mechanical plaque control; however, their antimicrobial efficacy varies depending on composition and mechanism of action. The aim of this study was to comparatively evaluate the antimicrobial and antibiofilm activities of four commercially available dental products (Corsodyl, Ozosan, HybenX, and Elugel) against a broad spectrum of oral microorganisms. This in vitro study included Gram-positive and Gram-negative bacterial strains, comprising both reference strains and clinical isolates, as well as *Candida albicans*. Antimicrobial activity was assessed using the disc diffusion assay, while antibiofilm activity was evaluated using a crystal violet microplate assay. All experiments were performed in triplicate. Statistical analysis was conducted using two-way ANOVA followed by Tukey’s post hoc test (*p* < 0.05). All tested products exhibited antimicrobial activity. Inhibition zones ranged from 9 to 56 mm for Gram-positive bacteria, 12 to 38 mm for Gram-negative bacteria, and 13 to 43 mm for *Candida albicans*. Two-way ANOVA revealed a significant effect of the dental product (*p* < 0.001), while incubation time was not significant (*p* > 0.05). HybenX showed the highest antimicrobial efficacy, while chlorhexidine-based products demonstrated consistent activity. Antibiofilm inhibition exceeded 80% for several strains. Dental antiseptics exhibit significantly different antimicrobial and antibiofilm profiles, highlighting the importance of appropriate product selection in oral biofilm control.

## 1. Introduction

The human oral cavity hosts a complex microbial ecosystem composed of numerous bacterial and fungal species organized within structured polymicrobial biofilms. Dental plaque represents one of the most common biofilms and plays a key role in the development of oral diseases such as dental caries, gingivitis, and periodontitis. These microbial communities are embedded in an extracellular polymeric matrix that facilitates microbial adhesion and interactions while also providing protection against environmental stressors, including antimicrobial agents, thereby contributing to increased microbial persistence [1].

Oral biofilm formation is a dynamic process involving microbial adhesion, coaggregation, and the development of multispecies communities. Early colonizers, such as Streptococcus species, facilitate the attachment of secondary microorganisms, leading to the formation of structured biofilms. As the biofilm matures, increased microbial interactions and matrix development enhance tolerance to antimicrobial agents, making biofilm-associated infections difficult to eradicate. Consequently, the control and disruption of dental biofilms remain essential objectives in preventive and therapeutic dental care [1,2,3,4].

Chemical antiseptics are widely used as adjunctive approaches to mechanical plaque removal in order to reduce microbial load and prevent biofilm formation. Among these agents, chlorhexidine is considered one of the most widely used oral antiseptics because of its broad-spectrum antimicrobial activity against Gram-positive and Gram-negative bacteria as well as fungi. Chlorhexidine-containing formulations such as mouthwashes and topical gels are frequently recommended for plaque control and for the management of periodontal and peri-implant diseases [5]. The antimicrobial activity of chlorhexidine is mainly attributed to its ability to disrupt microbial cell membranes and precipitate intracellular components, ultimately leading to microbial cell death.

Despite its extensive clinical use, recent studies have raised concerns regarding the potential impact of chlorhexidine on the oral microbiome. Investigations have demonstrated that chlorhexidine mouthwash may induce significant alterations in the composition of the oral microbial community, affecting the balance between commensal and pathogenic microorganisms. Moreover, experimental studies have shown that even sub-minimum inhibitory concentrations of chlorhexidine can influence the development and structural integrity of multispecies biofilms, suggesting that its antimicrobial activity may also affect microbial interactions within biofilm communities [4,6]. These findings have stimulated the search for complementary antimicrobial strategies capable of disrupting biofilms while maintaining the balance of the oral microbiota.

In recent years, alternative strategies targeting biofilm disruption have gained increasing attention. One such approach involves the use of desiccating agents capable of rapidly dehydrating the biofilm matrix and facilitating the removal of organic debris from oral surfaces. These agents, typically based on sulfonated phenolic compounds with strong hygroscopic properties, act by inducing rapid dehydration and denaturation of the extracellular biofilm matrix, leading to structural collapse and increased susceptibility of embedded microorganisms. Experimental studies have demonstrated that such desiccating compounds exhibit significant antimicrobial activity against a broad range of bacterial and fungal pathogens and may contribute to the disruption and mechanical removal of biofilm-associated microorganisms [7,8].

Another emerging antimicrobial approach in dentistry involves the use of ozone-based formulations. Ozone is a highly reactive oxidizing agent capable of damaging microbial cell membranes, proteins, and nucleic acids, resulting in rapid microbial inactivation [9]. Several experimental studies have reported that ozone can effectively reduce the viability of oral microorganisms and inhibit biofilm formation. For example, gaseous ozone has been shown to significantly reduce *Enterococcus faecalis* biofilms in vitro, while ozonated oils have demonstrated antimicrobial activity against Candida species and cariogenic bacteria such as *Streptococcus mutans* [10,11]. Furthermore, recent studies have suggested that ozone may enhance antimicrobial activity when combined with conventional oral antiseptics such as chlorhexidine or fluoride, highlighting its potential as an adjunctive antimicrobial strategy in oral care [12].

In addition to classical oral pathogens such as *Streptococcus mutans*, which plays a major role in dental caries development, the oral microbiome may also include opportunistic microorganisms such as *Staphylococcus aureus*, *Staphylococcus epidermidis*, and *Candida albicans*. These microorganisms may participate in the formation of polymicrobial biofilms and may contribute to oral infections, particularly in immunocompromised individuals or in the presence of dysbiosis. The emergence of antibiotic-resistant strains, including methicillin-resistant *Staphylococcus aureus* (MRSA), further highlights the need for effective antimicrobial strategies capable of controlling diverse microbial populations.

Despite the wide range of antiseptic products available for oral care, comparative studies evaluating the antimicrobial and antibiofilm activities of different commercially available dental products remain limited. Many investigations have focused on individual antimicrobial agents or specific microorganisms, whereas oral biofilms consist of complex polymicrobial communities that include Gram-positive bacteria, Gram-negative bacteria, and fungi [1,2,3,4]. Therefore, further research is needed to better understand the antimicrobial potential of commonly used dental products against diverse microbial species.

Therefore, the aim of the present study was to comparatively evaluate the antimicrobial and antibiofilm activities of four commercially available dental products frequently used in oral care: Corsodyl, Ozosan, HybenX, and Elugel. By assessing their activity against a diverse panel of Gram-positive bacteria, Gram-negative bacteria, and Candida albicans, including both reference strains and clinical isolates, this study provides a clinically relevant comparative perspective on the efficacy of commonly used oral antiseptics in biofilm control.

The null hypothesis of the present study was that no significant differences exist among the tested dental products in terms of antimicrobial activity and biofilm formation inhibition against the investigated microbial strains.

## 2. Results

### 2.1. Antimicrobial Activity Against Gram-Positive Bacterial Strains

The antimicrobial activity of the four commercial dental products was evaluated against eight Gram-positive bacterial strains, including reference strains and clinical isolates. The results, expressed as inhibition zone diameters (mm) measured after 24 h and 48 h of incubation, are summarized in Table 1.

Among the tested dental products, HybenX consistently produced the largest inhibition zones against most Gram-positive strains, indicating a strong antibacterial effect. Moderate antimicrobial activity was observed for Corsodyl and Elugel, while Ozosan showed variable inhibition depending on the tested strain.

Across all Gram-positive strains tested, measurable inhibition zones were observed for all dental products, with variations depending on the bacterial species, incubation time, and product tested. In general, inhibition zones recorded at 48 h were comparable to or slightly higher than those observed at 24 h. This observation is consistent with the diffusion of antimicrobial compounds through the culture medium, leading to increased bacterial exposure over time.

For *Staphylococcus aureus MRSA* (ATCC 43300), inhibition zones ranged between approximately 14 and 43 mm, depending on the tested product and incubation time. Similar patterns were observed for the clinical MRSA isolate, as well as for *Staphylococcus aureus* ATCC 29213 and its corresponding clinical isolate, with inhibition zone diameters generally maintained or moderately increased after 48 h of incubation.

*Staphylococcus epidermidis* ATCC 14990 exhibited inhibition zones ranging from approximately 16 to 41 mm, while *Streptococcus mutans* ATCC 35668 showed inhibition zones between approximately 14 and 36 mm. In contrast, *Streptococcus sanguinis* ATCC 10556 demonstrated lower inhibition zone diameters overall, with values ranging from approximately 9 to 14 mm.

For *Lacticaseibacillus casei* ATCC 393, inhibition zones varied widely among the tested products, with values ranging from approximately 9 to 56 mm. The antibacterial reference agent gentamicin produced inhibition zones within the expected range for all Gram-positive strains tested.

A comparison between reference strains and corresponding clinical isolates of *Staphylococcus aureus* revealed generally similar susceptibility patterns across the tested dental products. Although minor variations in inhibition zone diameters were observed, these differences were not consistent across all products, indicating comparable antimicrobial responses under the tested conditions.

### 2.2. Antimicrobial Activity Against Gram-Negative Bacterial Strains

The antimicrobial activity of the dental products against Gram-negative bacteria was evaluated using three strains, including one reference strain and one clinical isolate of *Escherichia coli*, as well as *Salmonella typhimurium* ATCC 14028. The results are presented in Table 2.

Similar trends were observed for Gram-negative bacteria, where HybenX produced the largest inhibition zones, particularly against *Salmonella typhimurium*. The other tested products demonstrated moderate antimicrobial effects with relatively stable inhibition zones between 24 h and 48 h.

For *Escherichia coli* ATCC 25922, inhibition zone diameters ranged from approximately 13 to 35 mm, depending on the tested product and incubation time. Comparable inhibition patterns were observed for the clinical *E. coli* isolate, with inhibition zones generally maintained or slightly increased after 48 h.

*Salmonella typhimurium* ATCC 14028 exhibited inhibition zones ranging from approximately 12 to 38 mm across the tested dental products. As observed for Gram-positive bacteria, inhibition zones recorded at 48 h were generally similar to or larger than those measured at 24 h.

Gentamicin, used as the antibacterial reference agent, produced consistent inhibition zones for all Gram-negative strains tested.

A similar comparison between the reference strain and clinical isolate of *Escherichia coli* demonstrated comparable susceptibility patterns across the evaluated dental products. Slight differences in inhibition zone diameters were observed; however, no consistent trend suggesting altered susceptibility in the clinical isolate was identified.

### 2.3. Antifungal Activity Against Candida albicans

The antifungal activity of the dental products was assessed against *Candida albicans* ATCC 10231, with inhibition zones measured after 48 h and 72 h of incubation. The results are presented in Table 3.

HybenX also demonstrated the strongest antifungal activity against *Candida albicans*, producing inhibition zones considerably larger than those observed for the other tested dental products.

All tested dental products exhibited detectable antifungal activity against *Candida albicans*, with inhibition zone diameters ranging from approximately 13 to 43 mm. Inhibition zones recorded after 72 h were generally comparable to or slightly higher than those measured after 48 h. Amphotericin B, used as the reference antifungal agent, produced inhibition zones within the expected range.

### 2.4. Inhibition of Biofilm Formation

**Figure 1:**
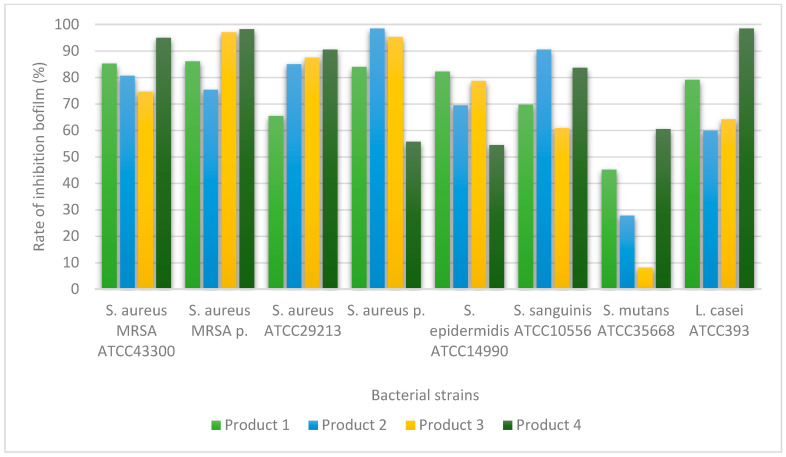
Inhibition of biofilm formation by the tested dental products against Gram-positive bacterial strains. Values represent mean values obtained from three independent experiments; variability is expressed as standard deviation (SD). OD570 values are proportional to biofilm biomass. Product 1 (Corsodyl), Product 2 (Ozosan), Product 3 (HybenX), and Product 4 (Elugel.).

**Figure 2:**
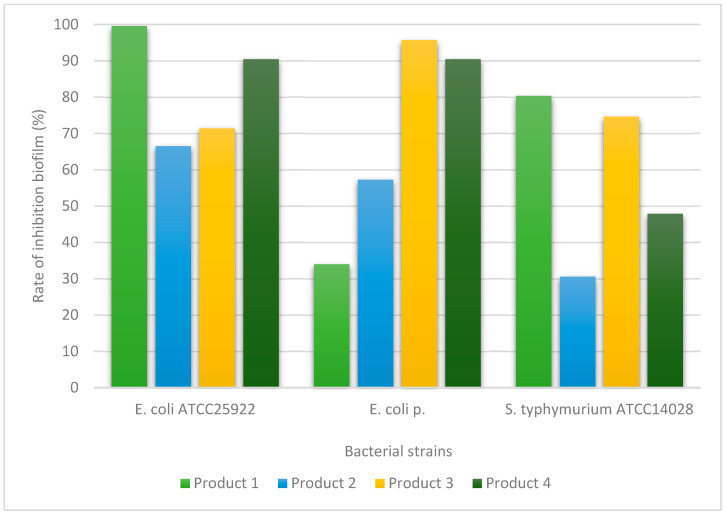
Inhibition of biofilm formation by the tested dental products against Gram-negative bacterial strains. Values represent mean values obtained from three independent experiments; variability is expressed as standard deviation (SD). OD570 values are proportional to biofilm biomass. Product 1 (Corsodyl), Product 2 (Ozosan), Product 3 (HybenX), and Product 4 (Elugel).

**Figure 3:**
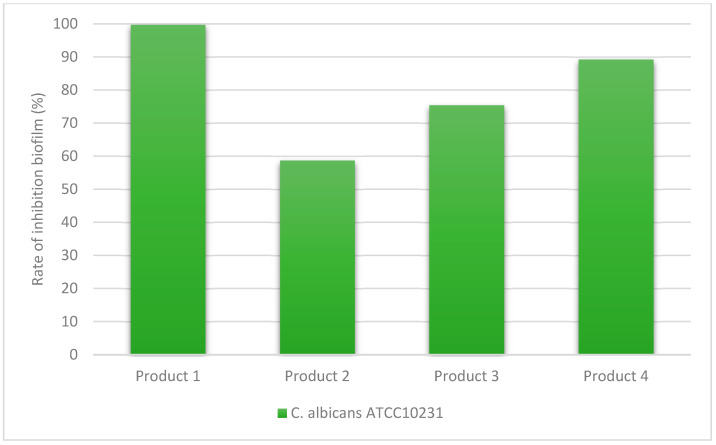
Inhibition of biofilm formation by the tested dental products against *Candida albicans*. Values represent mean ± SD (OD570) obtained from three independent experiments. OD570 values are proportional to biofilm biomass.

The ability of the dental products to inhibit biofilm formation was evaluated using a spectrophotometric assay. The results are presented graphically in Figure 1, Figure 2 and Figure 3 and demonstrate variations in antibiofilm activity depending on the tested product and the microbial strain.

Differences in the inhibition of biofilm formation were observed among the tested dental products for Gram-positive bacteria (Figure 1).

In the case of Gram-positive bacteria, relatively high levels of biofilm formation inhibition were observed, with inhibition rates exceeding 50% for three of the tested products, with a single exception noted for the *S. mutans* ATCC 35668 strain. For *S. mutans* ATCC 35668, Elugel was the only product that achieved a biofilm inhibition rate above 60%. Corsodyl exhibited biofilm inhibition rates exceeding 80% against *Staphylococcus aureus* ATCC 43300 (MRSA), *Staphylococcus aureus*, and *Staphylococcus epidermidis* ATCC 14990. For Ozosan, inhibition rates above 80% were observed for *Staphylococcus aureus* ATCC 43300 (MRSA), clinical isolates of MRSA, and *Staphylococcus aureus* ATCC 29213, whereas the highest level of biofilm formation was recorded for *Lacticaseibacillus casei* ATCC 393. HybenX demonstrated biofilm inhibition rates exceeding 80% against *Staphylococcus aureus* ATCC 29213, clinical isolates of MRSA, and *Staphylococcus aureus*. The dental product Elugel exhibited biofilm inhibition rates above 80% for *Staphylococcus aureus* ATCC 43300 (MRSA), clinical isolates of MRSA, *Staphylococcus aureus* ATCC 29213, *Streptococcus sanguinis* ATCC 10556, and *Lacticaseibacillus casei* ATCC 393.

For Gram-negative bacteria, variations in antibiofilm activity were also observed among the tested dental products, with differences depending on the bacterial species analyzed (Figure 2).

In the case of Gram-negative bacteria, the inhibitory effect on biofilm formation for Corsodyl was observed in *Escherichia coli* ATCC 25922 and *Salmonella typhimurium* ATCC 14028, with biofilm inhibition rates ranging between 80% and 100%. For Ozosan, a pronounced antibiofilm effect was identified for Escherichia coli ATCC 25922. HybenX demonstrated consistent antibiofilm activity across all three Gram-negative bacterial strains included in the study. Elugel exhibited effective biofilm inhibition against *Escherichia coli* ATCC 25922 and the clinical isolate of *Escherichia coli*.

A similar pattern was observed for the fungal strain, where the tested dental products showed variable inhibition of biofilm formation (Figure 3).

All experiments were performed in triplicate, and the results are expressed as mean ± standard deviation (SD).

Effective inhibition of biofilm formation in *Candida albicans* was observed for three of the four tested dental products. A decreasing trend in antibiofilm efficacy among the tested formulations was noted in the following order: Corsodyl > Elugel > HybenX.

### 2.5. Inferential Statistical Analysis

The two-way ANOVA revealed a highly significant main effect of the dental product on antimicrobial activity (F (3, 352) = 185.42, *p* < 0.001), with a large effect size (partial η^2^ = 0.61), indicating substantial differences in inhibition zone diameters among the tested formulations. In contrast, the main effect of incubation time was not statistically significant (F (1, 352) = 2.13, *p* = 0.145, partial η^2^ = 0.006), suggesting that antimicrobial activity remained relatively stable between 24 h and 48 h. The interaction between time and product was also not statistically significant (F (3, 352) = 1.87, *p* = 0.134, partial η^2^ = 0.01), indicating that the relative efficacy of the tested products was consistent across both time points.

Post hoc analysis using Tukey’s Honestly Significant Difference (HSD) test demonstrated that HybenX exhibited significantly higher antimicrobial activity compared to all other tested products (mean differences ranging between 18.2 and 26.5 mm, all *p* < 0.001). Ozosan showed significantly greater inhibition zone diameters than Corsodyl (mean difference = 3.1 mm, *p* = 0.012) and Elugel (mean difference = 2.8 mm, *p* = 0.018), while no statistically significant differences were observed between Corsodyl and Elugel (mean difference = 0.4 mm, *p* = 0.421).

Overall, these results confirm that the type of dental product represents the primary determinant of antimicrobial efficacy, whereas the influence of incubation time is limited under the tested experimental conditions.

## 3. Discussion

The findings of the present study do not support the null hypothesis, as the tested dental products exhibited distinct antimicrobial and antibiofilm profiles.

The present study comparatively evaluated the antimicrobial and antibiofilm activities of four commercially available dental products frequently used in oral care, including two chlorhexidine-based formulations (Corsodyl and Elugel), an ozone-based product (Ozosan), and a desiccating agent (HybenX). The results demonstrated that all tested products exhibited measurable antimicrobial activity against the investigated microorganisms, although considerable differences were observed in inhibition zone diameters depending on the product and microbial strain. These findings are consistent with previous studies showing that topical antimicrobial agents may differ in their efficacy because of differences in composition, mechanism of action, and diffusion behavior in vitro [13].

The comparison between reference strains and clinical isolates demonstrated generally similar susceptibility patterns across the tested dental products. Although slight variations in inhibition zone diameters were observed, no consistent trend indicating increased resistance in clinical isolates was identified. These findings suggest that the antimicrobial activity of the evaluated dental formulations is maintained against both laboratory reference strains and clinically derived microorganisms, supporting their potential relevance in real-world conditions.

These findings support the potential selection of specific formulations according to their clinical indications, including plaque control, periodontal therapy, and biofilm disruption strategies.

The increased tolerance of microorganisms organized within biofilms represents a major challenge in oral antimicrobial therapy [1]. Biofilms are structured microbial communities embedded within an extracellular polymeric matrix that acts as a protective barrier against environmental stressors and antimicrobial compounds. This matrix can limit the penetration of antimicrobial agents and contribute to the survival of microorganisms within the biofilm architecture [1,14]. Consequently, even antiseptic agents with broad antimicrobial activity may exhibit reduced effectiveness against mature biofilms compared with planktonic bacterial cells [7]. These characteristics highlight the importance of evaluating both antimicrobial and antibiofilm activities when assessing dental products intended for clinical use.

Among the tested dental products, the desiccating agent HybenX produced the largest inhibition zones against most microbial strains. This pronounced antimicrobial activity may be related to its reported mechanism of action, which involves desiccation and denaturation of the biofilm matrix. However, it should be noted that the present study design does not allow for direct investigation of this mechanism. The antimicrobial activity was assessed using an agar-based diffusion assay under hydrated conditions, which does not replicate the structural complexity of biofilms or permit evaluation of dehydration-mediated effects. Therefore, the proposed mechanism should be interpreted as a hypothesis supported by existing literature rather than a direct observation from the present study. Such effects have been reported in the literature; however, they were not directly investigated in the present study [7,15]. Previous studies have suggested that desiccating agents may facilitate the disruption of biofilm architecture and promote the removal of microbial deposits from oral surfaces.

The chlorhexidine-based formulations Corsodyl and Elugel also demonstrated consistent antimicrobial activity against the tested microorganisms. Chlorhexidine remains one of the most widely used antiseptic agents in dentistry due to its broad antimicrobial spectrum and its substantivity in the oral cavity. The antimicrobial action of chlorhexidine primarily involves disruption of bacterial cell membranes and precipitation of intracellular components, ultimately leading to microbial cell death [5,16]. Numerous studies have confirmed the effectiveness of chlorhexidine against a wide range of oral pathogens, including *Streptococcus mutans*, *Staphylococcus aureus*, and *Candida albicans* [5]. Nevertheless, the moderate inhibition zones observed in the present study may reflect the intrinsic resistance of biofilm-associated microorganisms and potential limitations related to product formulation or diffusion properties.

The ozone-based product Ozosan also exhibited antimicrobial activity against both bacterial and fungal strains, although its inhibitory effect was generally lower than that observed for the desiccating agent. Ozone is a powerful oxidizing agent capable of damaging microbial cell membranes, proteins, and nucleic acids through oxidative reactions [17,18]. Several studies have demonstrated that ozone can effectively reduce the viability of oral microorganisms and may inhibit biofilm formation [19]. Ozone-based therapies have therefore been proposed as adjunctive antimicrobial strategies in dentistry, particularly for the management of dental caries, periodontal infections, and oral microbial dysbiosis. However, the antimicrobial efficacy of ozone formulations may depend on factors such as concentration, exposure time, and the physical state of the ozone-containing product [20,21].

The antimicrobial activity of the tested dental products was generally more pronounced against Gram-positive bacterial strains compared to Gram-negative ones. This difference in antimicrobial efficacy may be explained by the structural differences in bacterial cell envelopes. Gram-positive bacteria possess a thick peptidoglycan layer and lack an outer membrane, which facilitates the penetration of active compounds from the tested dental products and enhances their antibacterial action. In most cases, these active compounds act by disrupting the integrity of the cell wall, leading to cell lysis and subsequent impairment of cellular metabolic activity, including the inhibition of enzymes involved in the synthesis of essential cellular components. In contrast, Gram-negative bacteria are characterized by the presence of an outer membrane rich in lipopolysaccharides, located external to a relatively thin peptidoglycan layer. This outer membrane acts as a selective barrier that limits the penetration of antimicrobial agents, thereby reducing their effectiveness. These findings are consistent with previously reported studies investigating compounds with antimicrobial potential, which have similarly demonstrated higher susceptibility of Gram-positive bacteria compared to Gram-negative bacteria [22,23].

The results of the present study regarding biofilm formation inhibition highlighted the superior efficacy of Elugel, which nearly completely suppressed biofilm formation in MRSA strains. All tested products exhibited antibiofilm activity, with more pronounced effects observed in Gram-positive bacteria, likely due to the structural characteristics of their cell wall and the increased accessibility of active antimicrobial compounds from the tested dental formulations. MRSA strains and clinical isolates appeared to be more susceptible to biofilm inhibition, which may be related to their enhanced biofilm-forming capacity, but also to the presence of multiple extracellular targets that are accessible to the active compounds present in the tested dental products. These findings are consistent with previously published studies investigating compounds with antimicrobial and antibiofilm potential, which have similarly reported enhanced susceptibility of Gram-positive bacteria and biofilm-forming strains to such agents [24,25].

The antifungal activity observed against *Candida albicans* is also noteworthy, as this opportunistic fungal pathogen is frequently associated with oral candidiasis and polymicrobial biofilm infections. *Candida* species are capable of forming complex biofilms on oral tissues and dental materials, which can increase resistance to antifungal agents and host immune responses. Previous studies have shown that oxidizing agents and other antimicrobial compounds may inhibit the growth of *Candida albicans* and interfere with fungal biofilm development [26,27].

In addition, the antimicrobial and antibiofilm effects observed in the present study appear to be more pronounced compared with those reported for natural formulations based on essential oils, which generally exhibit moderate biofilm inhibition rates below 30% depending on the microbial strain and experimental conditions [28]. In contrast, the dental products evaluated in this study demonstrated substantially higher antibiofilm activity, frequently exceeding 80% across multiple strains. These differences may be attributed to the distinct mechanisms of action of the active compounds, as chlorhexidine-based formulations and desiccating agents induce membrane disruption, protein denaturation, and biofilm matrix destabilization, whereas essential oils typically exert less potent antimicrobial effects [29,30].

The antibiofilm assays performed in the present study further demonstrated that the tested dental products were capable of inhibiting microbial biofilm formation to varying degrees. The ability to prevent or disrupt biofilm development represents a critical property for oral antiseptic agents, since biofilm formation is a key factor in the pathogenesis of many oral diseases, including dental caries and periodontal infections. Previous investigations have emphasized that effective oral antimicrobial strategies should target not only planktonic microbial cells but also the structural components of biofilms [31]. Therefore, the antibiofilm effects observed for the tested dental products may have important implications for their potential clinical application in the prevention and management of oral microbial infections.

Moreover, these findings may support the development of personalized preventive and therapeutic approaches in oral healthcare. The selection of specific antimicrobial formulations could be adapted according to individual patient characteristics, including oral microbiome composition, presence of systemic conditions associated with oral health, and overall risk profile for biofilm-associated diseases. In this context, the use of chlorhexidine-based formulations or alternative agents may be tailored as part of individualized oral hygiene protocols, particularly in patients requiring enhanced plaque control or adjunctive home-care strategies.

In addition to conventional antiseptic agents, emerging strategies such as bioactive coatings and antimicrobial peptides have gained increasing attention for the prevention and control of oral biofilms. These approaches may provide targeted antimicrobial activity, including against anaerobic bacteria, while potentially improving biocompatibility and reducing disruption of the oral microbiome. Compared to traditional antiseptics, which exhibit broad-spectrum activity, these novel strategies may allow more selective modulation of microbial communities. However, their clinical application remains limited, and further research is required to establish their efficacy and long-term safety in oral healthcare settings.

Although the present study provides relevant information regarding the antimicrobial and antibiofilm activities of several commercially available dental products, certain limitations should be acknowledged. First, the experiments were performed under controlled in vitro conditions, which cannot fully reproduce the complex ecological environment of the oral cavity. In vivo oral biofilms are influenced by multiple factors, including saliva composition, host immune responses, nutrient availability, and interactions among numerous microbial species within polymicrobial communities. In addition, the antimicrobial activity was evaluated using the disc diffusion assay, a method that may be influenced not only by the antimicrobial potency of the tested compounds but also by their physicochemical properties, such as viscosity and diffusion capacity in agar media. Furthermore, the crystal violet assay primarily evaluates early biofilm formation and does not completely reflect the structural complexity and increased antimicrobial tolerance observed in mature biofilms. Therefore, additional studies using more complex multispecies biofilm models and in vivo experimental approaches are necessary to further validate the antimicrobial potential of the tested dental products under clinical conditions [32].

Despite these limitations, the results of the present study provide useful insights into the antimicrobial potential of commonly used dental antiseptic products. The observed efficacy of the desiccating formulation further supports the relevance of matrix-targeting strategies [13].

Another limitation of the present study is the absence of minimum inhibitory concentration (MIC) and minimum bactericidal concentration (MBC) determinations, which could provide more precise quantitative information regarding the antimicrobial potency of the tested products. The disc diffusion assay used in this study offers a comparative evaluation of antimicrobial activity but may be influenced by diffusion characteristics of the formulations.

Furthermore, the antibiofilm assessment was performed using a single-species model, which does not fully replicate the structural and functional complexity of multispecies oral biofilms. In vivo oral biofilms involve complex microbial interactions that may influence antimicrobial susceptibility. Therefore, future studies employing multispecies biofilm models and complementary quantitative methods are necessary to further validate the present findings.

The antimicrobial activity assessed by the disc diffusion assay may be influenced not only by the intrinsic antimicrobial properties of the active compounds but also by the physicochemical characteristics of the tested formulations. Commercial dental products represent complex systems that include excipients such as solvents, thickeners, and stabilizing agents, which may affect the diffusion of active substances through the agar medium. Therefore, the diameter of inhibition zones should be interpreted as reflecting the overall antimicrobial performance of the formulation rather than the isolated activity of the active ingredient. This methodological aspect represents an inherent limitation of diffusion-based assays and should be considered when comparing products with different formulations.

Another aspect that should be considered is that the inferential statistical analysis was performed on pooled data across multiple microbial species in order to evaluate overall trends in antimicrobial activity. This approach allows for the identification of general differences between tested products. It may partially obscure variability in susceptibility between individual species and strains. To address this, the results were also presented in a stratified manner, with inhibition zone diameters reported separately for each microbial strain. Therefore, species-specific differences should be interpreted based on these detailed data.

## 4. Materials and Methods

### 4.1. Dental Products Evaluated

The antimicrobial and antibiofilm activities were investigated for four commercially available dental products routinely used in oral care. All products were tested in their original commercial formulations, without any prior modification, under sterile conditions (Table 4).

### 4.2. Microbial Strains

The antimicrobial activity of the dental products was assessed against a diverse panel of microorganisms, including both reference strains and clinical isolates, in order to ensure experimental robustness and clinical relevance. The tested panel comprised Gram-positive and Gram-negative bacterial strains, as well as a fungal strain.

The Gram-positive bacteria included the following reference strains: *Staphylococcus aureus* ATCC 43300 (MRSA), *Staphylococcus aureus* ATCC 29213, *Staphylococcus epidermidis* ATCC 14990, *Streptococcus sanguinis* ATCC 10556, *Streptococcus mutans* ATCC 35668, and *Lacticaseibacillus casei* ATCC 393. In addition, clinical isolates of MRSA and *Staphylococcus aureus* were included in the analysis. The Gram-negative strains comprised the reference strains *Escherichia coli* ATCC 25922 and *Salmonella typhimurium* ATCC 14028, as well as a clinical isolate of *Escherichia coli*. The antifungal activity was evaluated against *Candida albicans* ATCC 10231.

All reference strains were obtained from the culture collection of the Department of Cellular and Molecular Biology, Victor Babeș University of Medicine and Pharmacy, Timișoara, Romania. Clinical bacterial isolates were provided by the Microbiology Laboratory of the Pius Brînzeu Emergency Clinical Hospital, Timișoara, Romania.

### 4.3. Microbial Cultivation and Inoculum Preparation

Prior to antimicrobial testing, all microbial strains were cultivated under standardized conditions. Bacterial reference strains and clinical isolates were pre-cultured for 24 h at 37 °C in Trypticase Soy Broth Medium (TSB), while the fungal strain was pre-cultured for 48 h at 37 °C in Sabouraud Liquid Medium (SL).

Following incubation, the optical density (OD) of each microbial culture was measured at 600 nm using a BioTek Synergy H1 microplate reader (Agilent, Santa Clara, CA, USA). Based on the obtained values, appropriate dilutions were performed using the corresponding culture media in order to obtain standardized microbial suspensions adjusted to 0.5 McFarland standard, corresponding to approximately 1.5 × 10^8^ CFU/mL. The turbidity of the inoculum was verified using a McFarland densitometer (Grand-Bio, London, UK). These standardized microbial suspensions were subsequently used for all antimicrobial and antibiofilm assays [33].

### 4.4. Evaluation of Antimicrobial Activity by Disc Diffusion Assay

The antimicrobial activity of the tested dental products was evaluated using the disc diffusion assay, a widely employed method for assessing microbial susceptibility to antimicrobial agents. The method is based on the diffusion of the tested compounds from impregnated discs into a solid culture medium previously inoculated with a standardized microbial suspension, resulting in the formation of a concentration gradient around each disc [34,35,36].

Tryptic Soy Agar (TSA) was used for bacterial strains, while Sabouraud Dextrose Agar (SDA) was employed for the fungal strain. Culture media were prepared according to the manufacturers’ instructions and sterilized by autoclaving. After solidification, the agar surfaces were uniformly inoculated with the standardized microbial suspensions using sterile L-shaped spreaders. The inoculated plates were allowed to rest under a laminar flow hood for 15 min to ensure adequate absorption of the inoculum.

Sterile discs were subsequently placed on the agar surface and impregnated with the tested dental products. The plates were incubated at 37 °C, and antimicrobial activity was assessed by measuring the diameter of the inhibition zones surrounding each disc. Measurements were performed after 24 h and 48 h of incubation for bacterial strains, and after 48 h and 72 h for the fungal strain. Inhibition zones were recorded in millimeters using a calibrated ruler.

Due to the nature of the disc diffusion method, antimicrobial activity was evaluated based on inhibition zones around product-impregnated discs, and therefore a non-treated surface control was not applicable in this experimental setup.

Gentamicin (ROTI^®^ Antibiotic Discs Gentamicin, 10 µg; Carl Roth, Karlsruhe, Germany) was used as the reference antibacterial agent, while Amphotericin B (ROTI^®^ Antibiotic Discs Amphotericin B, 100 units; Carl Roth, Germany) served as the reference antifungal agent. These agents were selected as standard laboratory controls with well-established antimicrobial activity in disc diffusion assays, in order to validate microbial susceptibility and ensure the reliability of the experimental conditions, rather than to provide a direct clinical comparison with the tested dental products.

Inhibition zones were measured in millimeters. All experiments were performed in triplicate, and results were expressed as mean ± standard deviation.

### 4.5. Determination of Biofilm Formation Inhibition

The antibiofilm activity of the dental products was evaluated using a modified crystal violet assay, following the methodological adaptations described in recent studies [22,23]. Briefly, 100 µL of standardized bacterial inoculum was added to each well of a sterile 96-well microplate, followed by the addition of 50 µL of each tested dental product. The plates were incubated for 24 h at 37 °C to allow biofilm formation.

The experiments were performed using multiple 96-well microplates across independent replicates, rather than a single plate, in order to ensure reproducibility and avoid potential plate-specific bias.

After incubation, the wells were gently washed twice with sterile 0.9% NaCl solution to remove non-adherent cells and subsequently dried at 37 °C. Biofilms were stained with 200 µL of 0.4% crystal violet solution for 1 h at 37 °C. Excess dye was removed by washing with tap water, and the bound stain was solubilized by adding 200 µL of 30% acetic acid to each well, allowing it to act for 30 min.

Absorbance was measured at 570 nm using the BioTek Synergy H1 microplate reader. Untreated wells containing microbial inoculum without the addition of dental products were used as negative controls to provide a baseline for biofilm formation. The antibiofilm effect of the tested products was expressed relative to these controls (ODtreated/ODcontrol).

### 4.6. Statistical Analysis

Statistical analysis was performed using the raw data obtained from triplicate experiments (n = 3 per condition), allowing for an accurate estimation of intra-group variability. Inhibition zone diameters were considered continuous variables and were expressed as mean ± standard deviation (SD). A two-way analysis of variance (two-way ANOVA) was applied to evaluate the effects of incubation time (24 h vs. 48 h) and dental product (Corsodyl, Ozosan, HybenX, and Elugel) on antimicrobial activity, while also assessing the interaction between these two factors. Prior to applying the two-way ANOVA, data distribution and model residuals were assessed for approximate normality, and homogeneity of variances was evaluated. Although minor deviations were observed in some cases, the analysis was considered sufficiently robust for exploratory purposes. The analysis was conducted across all experimental conditions, considering each measurement was treated as an independent observation within the balanced experimental design for comparative purposes. When statistically significant differences were identified, post hoc multiple comparisons were performed using Tukey’s Honestly Significant Difference (HSD) test in order to compare all product pairs. Statistical significance was set at *p* < 0.05. All statistical analyses were performed using IBM SPSS Statistics software (version 30.0.0, IBM Corp., Armonk, NY, USA).

Each experimental condition (microbial strain × dental product) was tested in triplicate (n = 3), resulting in a total of 96 measurements (3 replicates × 4 products × 8 microbial strains). This balanced design allowed comparative evaluation across all tested conditions.

## 5. Conclusions

The present study demonstrates that the tested dental formulations exhibit distinct antimicrobial and antibiofilm profiles against a broad range of oral microorganisms, including Gram-positive and Gram-negative bacteria and *Candida albicans*. The desiccating agent showed the highest overall efficacy, particularly in biofilm inhibition, while chlorhexidine-based formulations provided consistent antimicrobial activity. The ozone-based formulation displayed moderate effects, supporting its role as a complementary approach.

The variability observed among products and microbial strains highlights the importance of selecting antimicrobial agents according to specific clinical and microbiological contexts, supporting a more individualized approach to oral biofilm management. However, given the in vitro design, further studies using complex biofilm models and clinical settings are required to confirm the translational relevance of these findings.

These findings have potential clinical implications, suggesting that the selection of dental antiseptic products may be guided by their specific antimicrobial and antibiofilm profiles, depending on the clinical context, including plaque control, periodontal therapy, and targeted biofilm management.

## Figures and Tables

**Table 1:** Inhibition zone diameters (mm) produced by the tested dental products against Gram-positive bacterial strains after 24 h and 48 h of incubation (mean ± SD).

Bacterial Strain	Tested Product	24 h	48 h
*S. aureus MRSA* ATCC43300	a	14.33 ± 1.03	15 ± 0.89
	b	16 ± 0.89	16.66 ± 1.36
	c	39.66 ± 2.25	42.66 ± 1.03
	d	14.66 ± 0.51	15.33 ± 0.51
	GEN	13 ± 0.89	13.66 ± 0.51
*S. aureus MRSA* p.	a	18 ± 0.89	18 ± 0.89
	b	13.66 ± 3.14	14.66 ± 2.87
	c	34 ± 1.78	35.33 ± 1.03
	d	17.66 ± 0.51	18 ± 0
	GEN	12.33 ± 0.51	12.66 ± 1.03
*S. aureus* ATCC29213	a	18.66 ± 0.51	18.66 ± 0.51
	b	17.66 ± 2.58	18.66 ± 2.73
	c	34.33 ± 7.60	35.66 ± 6.83
	d	17 ± 1.54	18.33 ± 2.250
	GEN	15 ± 0.894	15.666 ± 0.516
*S. aureus* p.	a	14 ± 0.89	15.33 ± 1.03
	b	13.33 ± 2.7	14 ± 1.78
	c	32 ± 4.73	32.66 ± 4.92
	d	14.33 ± 1.36	14.33 ± 1.36
	GEN	14.33 ± 1.36	15.33 ± 0.51
*S. epidermidis* ATCC14990	a	21 ± 1.54	21 ± 1.54
	b	16.33 ± 1.36	18.33 ± 0.51
	c	38.66 ± 2.73	40.66 ± 4.50
	d	21.66 ± 0.51	21.66 ± 0.51
	GEN	15.66 ± 0.51	16.33 ± 0.51
*S. sanguinis* ATCC10556	a	8.66 ± 0.51	9 ± 0.89
	b	9.66 ± 0.51	10 ± 0
	c	13.66 ± 0.51	14.33 ± 0.51
	d	10.33 ± 0.51	10.66 ± 0.51
	GEN	16.33 ± 0.51	16.66 ± 0.51
*S. mutans* ATCC35668	a	14 ± 0.89	15.33 ± 0.51
	b	16.33 ± 1.03	16.66 ± 0.51
	c	34.66 ± 3.72	36.33 ± 3.61
	d	15.33 ± 1.03	17 ± 2.36
	GEN	15.33 ± 0.51	16 ± 0
*L. casei* ATCC393	a	13.33 ± 1.03	14.33 ± 1.032
	b	28.66 ± 7.44	29 ± 7.09
	c	56 ± 4.73	56 ± 4.73
	d	9.66 ± 1.03	10.66 ± 1.03
	GEN	13.33 ± 1.86	14 ± 0.89

Abbreviations: a, Corsodyl; b, Ozosan; c, HybenX; d, Elugel; GEN, gentamicin; p., clinical isolate.

**Table 2:** Inhibition zone diameters (mm) produced by the tested dental products against Gram-negative bacterial strains after 24 h and 48 h of incubation (mean ± SD).

Bacterial Strain	Tested Product	24 h	48 h
*E. coli* ATCC25922	a	13.33 ± 0.51	14 ± 0
	b	17.33 ± 1.86	17.33 ± 1.86
	c	32 ± 8.19	35.33 ± 7.44
	d	14 ± 0.89	14.33 ± 1.36
	GEN	16.33 ± 0.51	16.66 ± 0.51
*E. coli* p.	a	13.66 ± 0.51	13.66 ± 0.51
	b	15.66 ± 1.03	16.33 ± 1.36
	c	32.33 ± 5.24	34 ± 4.73
	d	14.66 ± 1.03	15 ± 0.89
	GEN	16.33 ± 1.03	16.66 ± 0.51
*S. typhimurium* ATCC14028	a	12.66 ± 0.51	16.66 ± 1.03
	b	17 ± 1.54	17.66 ± 1.36
	c	36.66 ± 0.51	38.33 ± 1.36
	d	12 ± 1.78	13 ± 0.89
	GEN	16.33 ± 0.51	16.66 ± 0.51

Abbreviations: a, Corsodyl; b, Ozosan; c, HybenX; d, Elugel; GEN, gentamicin; p., clinical isolate.

**Table 3:** Inhibition zone diameters (mm) produced by the tested dental products against *Candida albicans* ATCC 10231 after 48 h and 72 h of incubation (mean ± SD).

Fungal Strain	Tested Product	48 h	72 h
*C. albicans* ATCC10231	a	15 ± 1.54	15.333 ± 1.36
	b	28.66 ± 1.03	30 ± 0
	c	40 ± 5.36	42.666 ± 4.13
	d	13.66 ± 2.06	14 ± 2.36
	AP	13.66 ± 0.51	14 ± 0.89

Abbreviations: a, Corsodyl; b, Ozosan; c, HybenX; d, Elugel; AP, Amphotericin B (reference antifungal agent).

**Table 4:** Characteristics of the evaluated dental products and their antimicrobial mechanisms.

Product	Manufacturer (Country)	Formulation/Active Compound	Product Type	Mechanism of Action/Clinical Use
Corsodyl	Haleon Group (Weybridge, UK)	Chlorhexidine digluconate 0.2% (mouthwash)	Chlorhexidine-based antiseptic	Broad-spectrum antimicrobial activity; disrupts bacterial cell membranes; used for plaque control and periodontal infections
Ozosan	Sanipan S.r.l. (Rome, Italy)	Stabilized ozone/ozonized sunflower oil (gel)	Ozone-based product	Oxidative damage to microbial cells; antimicrobial and biofilm inhibition
HybenX	EPIEN Medical, Inc. (St. Paul, MN, USA)	Sulfonated phenolic compounds (gel)	Desiccating agent	Rapid dehydration and denaturation of biofilm matrix; removal of organic debris
Elugel	Pierre Fabre Oral Care (Castres, France)	Chlorhexidine-containing gel	Chlorhexidine-based antiseptic	Membrane disruption and protein precipitation; topical antimicrobial action

## Data Availability

The original contributions presented in this study are included in the article. Further inquiries can be directed to the corresponding author.

## Abbreviations

The following abbreviations are used in this manuscript:

ATCC	American Type Culture Collection
CFU	Colony Forming Units
MRSA	Methicillin-Resistant *Staphylococcus aureus*
OD	Optical Density
SD	Standard Deviation
TSB	Trypticase Soy Broth
TSA	Tryptic Soy Agar
SDA	Sabouraud Dextrose Agar
SL	Sabouraud Liquid Medium
